# Supplementary material for: The profile of lipid metabolites in urine of marmoset wasting syndrome
Source: PLoS One. 2020 Jun 23;15(6):e0234634. doi: 10.1371/journal.pone.0234634 (PMC7310677; doi:10.1371/journal.pone.0234634)
Supplement: S1 Table — PUFA, polyunsaturated free fatty acid; SRM, selected reaction monitoring; ALA, α-linoleic acid; LA, linoleic acid; DGLA, dihomo-γ-linolenic acid; AA, arachidonic acid; EPA, eicosapentaenoic acid; DHA, docosahexaenoic acid. (DOCX) [file pone.0234634.s001.docx]

**Table S1. Measured 141 types of lipid metabolites and 15 types of internal standards.**

| **No.** | **PUFA** | **Metabolites** | **Internal standard** | **SRM (m/z)** | | **Retention time** | **Mode**  **(+/-)** |
| --- | --- | --- | --- | --- | --- | --- | --- |
|  |  |  |  | **precursor** | **product** |  |  |
| 1 | ALA | 9-HOTrE | LTB_4_-d_4_ | 293.2 | 171.1 | 15.66 | (−) |
| 2 | ALA | 13-HOTrE | LTB_4_-d_4_ | 293.2 | 195.1 | 15.835 | (−) |
| 3 | LA | 12,13-DiHOME | LTB_4_-d_4_ | 313.2 | 183.1 | 14.46 | (−) |
| 4 | LA | 9,10-DiHOME | LTB_4_-d_4_ | 313.2 | 201.2 | 14.63 | (−) |
| 5 | LA | 13-HODE | 15-HETE-d_8_ | 295.2 | 195.1 | 16.6 | (−) |
| 6 | LA | 9-HODE | 15-HETE-d_8_ | 295.2 | 171.1 | 16.6 | (−) |
| 7 | LA | 9-HpODE | 15-HETE-d_8_ | 311.2 | 185.2 | 16.995 | (−) |
| 8 | LA | 13-KODE | 15-HETE-d_8_ | 293.2 | 113.1 | 17 | (−) |
| 9 | LA | 13-HpODE | 15-HETE-d_8_ | 311.2 | 113.1 | 17.01 | (−) |
| 10 | LA | 9-KODE | 12-HETE-d_8_ | 293.2 | 185.1 | 17.12 | (−) |
| 11 | LA | 12,13-EpOME | 5-HETE-d_8_ | 295.2 | 195.2 | 17.78 | (−) |
| 12 | LA | 9,10-EpOME | 5-HETE-d_8_ | 295.2 | 171.1 | 17.85 | (−) |
| 13 | DGLA | TXB_1_ | TXB_2_-d_4_ | 371.2 | 171.2 | 9.285 | (−) |
| 14 | DGLA | 8-*iso*-PGF_1α_ | PGF_2α_-d_4_ | 355.2 | 293.2 | 9.485 | (−) |
| 15 | DGLA | 8-*iso*-PGE_1_ | PGE_2_-d_4_ | 353.2 | 235.2 | 10.795 | (−) |
| 16 | DGLA | PGE_1_ | PGD_2_-d_4_ | 353.2 | 235.2 | 11.08 | (−) |
| 17 | DGLA | PGD_1_ | PGD_2_-d_4_ | 353.2 | 235.1 | 11.205 | (−) |
| 18 | DGLA | 8-*iso*-PGA_1_ | LTB_4_-d_4_ | 335.2 | 235.2 | 12.99 | (−) |
| 19 | DGLA | PGA_1_ | LTB_4_-d_4_ | 335.2 | 235.2 | 13.33 | (−) |
| 20 | DGLA | 15-HETrE | 5-HETE-d_8_ | 321.2 | 221.2 | 17.44 | (−) |
| 21 | AA | AA | AA-d_8_ | 303.2 | 303.2 | 20.32 | (−) |
| 22 | AA | tetranor-PGFM | tetranor-PGEM-d_6_ | 329.2 | 311.2 | 2.66 | (−) |
| 23 | AA | tetranor-PGEM | tetranor-PGEM-d_6_ | 327.2 | 309.2 | 3.02 | (−) |
| 24 | AA | tetranor-PGDM | tetranor-PGEM-d_6_ | 327.2 | 309.2 | 3.26 | (−) |
| 25 | AA | 20-hydroxy-PGF_2α_ | 6-keto-PGF_1α_-d_4_ | 369.2 | 325.2 | 5.43 | (−) |
| 26 | AA | 20-hydroxy-PGE_2_ | 6-keto-PGF_1α_-d_4_ | 367.2 | 287.2 | 5.58 | (−) |
| 27 | AA | 18-carboxy-dinor-LTB_4_ | 6-keto-PGF_1α_-d_4_ | 337.2 | 59.1 | 6.09 | (−) |
| 28 | AA | 13,14-dihydro-15-keto-tetranor-PGF_1β_ | 6-keto-PGF_1α_-d_4_ | 299.2 | 113.1 | 6.62 | (−) |
| 29 | AA | 2,3-dinor-8-*iso*-PGF_2α_ | 6-keto-PGF_1α_-d_4_ | 325.2 | 237.2 | 7.22 | (−) |
| 30 | AA | 13,14-dihydro-15-keto-tetranor-PGF_1α_ | 6-keto-PGF_1α_-d_4_ | 299.2 | 113.1 | 7.52 | (−) |
| 31 | AA | 6-keto-PGF_1α_ | 6-keto-PGF_1α_-d_4_ | 369.2 | 245.2 | 7.68 | (−) |
| 32 | AA | 13,14-dihydro-15-keto-tetranor-PGD_2_ | 6-keto-PGF_1α_-d_4_ | 297.2 | 109.1 | 7.9 | (−) |
| 33 | AA | 20-carboxy-LTB_4_ | 6-keto-PGF_1α_-d_4_ | 365.2 | 169.1 | 8.05 | (−) |
| 34 | AA | 20-hydroxy-LTB_4_ | 6-keto-PGF_1α_-d_4_ | 351.2 | 195.1 | 8.34 | (−) |
| 35 | AA | 13,14-dihydro-15-keto-tetranor-PGE_2_ | 6-keto-PGF_1α_-d_4_ | 297.2 | 109.1 | 8.59 | (−) |
| 36 | AA | 6,15-diketo-13,14-dihydro-PGF_1α_ | 6-keto-PGF_1α_-d_4_ | 369.2 | 113.1 | 8.87 | (−) |
| 37 | AA | TXB_2_ | TXB_2_-d_4_ | 369.2 | 195.1 | 9.465 | (−) |
| 38 | AA | iPF_2α_-IV | PGF_2α_-d_4_ | 353.2 | 127.1 | 9.07 | (−) |
| 39 | AA | 8-*iso*-15(R)-PGF_2α_ | PGF_2α_-d_4_ | 353.2 | 193.1 | 9.27 | (−) |
| 40 | AA | 8-*iso*-PGF_2α_ | PGF_2α_-d_4_ | 353.2 | 193.1 | 9.425 | (−) |
| 41 | AA | 11-beta-PGF_2a_ | PGF_2α_-d_4_ | 353.2 | 193.1 | 9.63 | (−) |
| 42 | AA | 5-iPF_2α_-VI | PGF_2α_-d_4_ | 353.2 | 115.1 | 9.81 | (−) |
| 43 | AA | 8-*iso*-15-keto-PGF_2α_ | PGF_2α_-d_4_ | 351.2 | 219.2 | 9.94 | (−) |
| 44 | AA | PGF_2α_ | PGF_2α_-d_4_ | 353.2 | 193.1 | 10.27 | (−) |
| 45 | AA | 8-*iso*-13,14-dihydro-15-keto-PGF_2α_ | PGF_2α_-d_4_ | 353.2 | 183.1 | 10.46 | (−) |
| 46 | AA | 8-*iso*-PGE_2_ | PGE_2_-d_4_ | 351.2 | 271.2 | 10.54 | (−) |
| 47 | AA | PGE_2_ | PGE_2_-d_4_ | 351.2 | 271.2 | 10.7 | (−) |
| 48 | AA | 11-dehydro-TXB_2_ | PGE_2_-d_4_ | 367.2 | 305.2 | 10.77 | (−) |
| 49 | AA | 15-keto-PGF_2α_ | PGE_2_-d_4_ | 351.2 | 219.2 | 10.815 | (−) |
| 50 | AA | 5S,14R-LXB_4_ | PGD_2_-d_4_ | 351.3 | 221.2 | 10.98 | (−) |
| 51 | AA | PGK_2_ | PGD_2_-d_4_ | 349.2 | 249.2 | 11.07 | (−) |
| 52 | AA | PGD_2_ | PGD_2_-d_4_ | 351.2 | 271.2 | 11.1 | (−) |
| 53 | AA | 11-beta-13,14-dihydro-15-keto-PGF_2α_ | PGD_2_-d_4_ | 353.2 | 183.1 | 11.28 | (−) |
| 54 | AA | 15-keto-PGE_2_ | PGD_2_-d_4_ | 349.2 | 113.1 | 11.285 | (−) |
| 55 | AA | 13,14-dihydro-15-keto-PGF_2α_ | PGD_2_-d_4_ | 353.2 | 113.1 | 11.72 | (−) |
| 56 | AA | 5S,6R-LXA_4_ | PGD_2_-d_4_ | 351.2 | 115.1 | 11.805 | (−) |
| 57 | AA | 13,14-dihydro-15-keto-PGE_2_ | PGD_2_-d_4_ | 351.2 | 175.1 | 11.895 | (−) |
| 58 | AA | 5S,6S-LXA4 | PGD_2_-d_4_ | 351.2 | 115.1 | 12.06 | (−) |
| 59 | AA | 13,14-dihydro-15-keto-PGD_2_ | PGD_2_-d_4_ | 351.2 | 207.2 | 12.54 | (−) |
| 60 | AA | 14,15-LTC_4_ | LTC_4_-d_5_ | 626.4 | 308.2 | 11.64 | (+) |
| 61 | AA | LTD_4_ | LTC_4_-d_5_ | 497.3 | 189.1 | 11.98 | (+) |
| 62 | AA | 14,15-LTE_4_ | LTC_4_-d_5_ | 440.3 | 301.2 | 12.135 | (+) |
| 63 | AA | LTC_4_ | LTC_4_-d_5_ | 626.4 | 308.2 | 12.61 | (+) |
| 64 | AA | LTE_4_ | LTC_4_-d_5_ | 440.3 | 189.1 | 12.72 | (+) |
| 65 | AA | LTF_4_ | LTC_4_-d_5_ | 569.4 | 251.2 | 12.84 | (+) |
| 66 | AA | 11-trans-LTC_4_ | LTC_4_-d_5_ | 626.4 | 189.1 | 12.92 | (+) |
| 67 | AA | 11-trans-LTE_4_ | LTC_4_-d_5_ | 440.3 | 189.1 | 12.99 | (+) |
| 68 | AA | 8-*iso*-PGA_2_ | LTB_4_-d_4_ | 333.2 | 271.2 | 12.875 | (−) |
| 69 | AA | PGA_2_ | LTB_4_-d_4_ | 333.2 | 271.2 | 13.02 | (−) |
| 70 | AA | PGJ_2_ | LTB_4_-d_4_ | 333.2 | 271.2 | 13.125 | (−) |
| 71 | AA | PGB_2_ | LTB_4_-d_4_ | 333.2 | 175.1 | 13.15 | (−) |
| 72 | AA | 8,12-*iso*-iPF_2α_-VI-1,5-lactone | LTB_4_-d_4_ | 337.2 | 265.2 | 13.8 | (+) |
| 73 | AA | 8,15-DiHETE | LTB_4_-d_4_ | 335.2 | 127.1 | 13.81 | (−) |
| 74 | AA | 6-trans-LTB_4_ | LTB_4_-d_4_ | 335.2 | 195.1 | 14 | (−) |
| 75 | AA | 5,15-DiHETE | LTB_4_-d_4_ | 335.2 | 173.1 | 14 | (−) |
| 76 | AA | LTB_4_ | LTB_4_-d_4_ | 335.2 | 195.1 | 14.12 | (−) |
| 77 | AA | 13,14-dihydro-15-keto PGJ_2_ | LTB_4_-d_4_ | 333.2 | 175.1 | 14.18 | (−) |
| 78 | AA | 12-keto-LTB_4_ | LTB_4_-d_4_ | 333.2 | 179.1 | 14.74 | (−) |
| 79 | AA | N-acetyl-LTE_4_ | LTB_4_-d_4_ | 480.3 | 333.2 | 14.955 | (−) |
| 80 | AA | 14,15-DHET | LTB_4_-d_4_ | 337.2 | 207.2 | 15.04 | (−) |
| 81 | AA | 12-HHT | LTB_4_-d_4_ | 279.2 | 179.1 | 15.155 | (−) |
| 82 | AA | 11,12-DHET | LTB_4_-d_4_ | 337.2 | 167.1 | 15.36 | (−) |
| 83 | AA | 8,9-DHET | LTB_4_-d_4_ | 337.2 | 127.1 | 15.54 | (−) |
| 84 | AA | 20-carboxy-AA | LTB_4_-d_4_ | 333.2 | 297.2 | 15.67 | (−) |
| 85 | AA | 5,6-DHET | LTB_4_-d_4_ | 337.2 | 145.1 | 15.82 | (−) |
| 86 | AA | 19-HETE | LTB_4_-d_4_ | 319.2 | 275.2 | 15.95 | (−) |
| 87 | AA | 15-deoxy-delta-12,14-PGJ_2_ | LTB_4_-d_4_ | 315.2 | 271.2 | 15.97 | (−) |
| 88 | AA | 20-HETE | 15-HETE-d_8_ | 319.2 | 245.3 | 16.07 | (−) |
| 89 | AA | 18-HETE | 15-HETE-d_8_ | 319.2 | 261.2 | 16.245 | (−) |
| 90 | AA | 17-HETE | 15-HETE-d_8_ | 319.2 | 247.2 | 16.36 | (−) |
| 91 | AA | 16-HETE | 15-HETE-d_8_ | 319.2 | 233.2 | 16.445 | (−) |
| 92 | AA | 15-HETE | 15-HETE-d_8_ | 319.2 | 219.2 | 16.93 | (−) |
| 93 | AA | 11-HETE | 12-HETE-d_8_ | 319.2 | 167.1 | 17.125 | (−) |
| 94 | AA | 8-HETE | 12-HETE-d_8_ | 319.2 | 155.1 | 17.2 | (−) |
| 95 | AA | 15-HpETE | 12-HETE-d_8_ | 335.2 | 113.1 | 17.27 | (−) |
| 96 | AA | 12-HETE | 12-HETE-d_8_ | 319.2 | 179.1 | 17.28 | (−) |
| 97 | AA | 9-HETE | 5-HETE-d_8_ | 319.2 | 123.1 | 17.315 | (−) |
| 98 | AA | 5-HETE | 5-HETE-d_8_ | 319.2 | 115.1 | 17.38 | (−) |
| 99 | AA | 12-HpETE | 5-HETE-d_8_ | 335.2 | 153.1 | 17.515 | (−) |
| 100 | AA | 12-KETE | 5-HETE-d_8_ | 317.2 | 153.1 | 17.56 | (−) |
| 101 | AA | 5,6-DHET-lactone | 5-HETE-d_8_ | 321.2 | 177.1 | 17.64 | (+) |
| 102 | AA | 5-HpETE | 5-HETE-d_8_ | 335.2 | 129.1 | 17.75 | (−) |
| 103 | AA | 14,15-EET | 5-HETE-d_8_ | 319.2 | 113.1 | 17.95 | (−) |
| 104 | AA | 5-KETE | OEA-d_4_ | 317.2 | 203.2 | 18.04 | (−) |
| 105 | AA | 11,12-EET | OEA-d_4_ | 319.2 | 167.1 | 18.26 | (−) |
| 106 | AA | 8,9-EET | OEA-d_4_ | 319.2 | 127.1 | 18.33 | (−) |
| 107 | AA | 5,6-EET | OEA-d_4_ | 319.2 | 191.1 | 18.34 | (−) |
| 108 | EPA | EPA | EPA-d_5_ | 301.2 | 257.2 | 19.415 | (−) |
| 109 | EPA | 8-*iso*-PGF_3α_ | 6-keto-PGF_1α_-d4 | 351.2 | 307.2 | 8.24 | (−) |
| 110 | EPA | PGF_3α_ | 6-keto-PGF_1α_-d_4_ | 351.2 | 193.1 | 9.04 | (−) |
| 111 | EPA | TXB_3_ | TXB_2_-d_4_ | 367.2 | 169.1 | 8.275 | (−) |
| 112 | EPA | PGE_3_ | PGF_2α_-d_4_ | 349.2 | 269.2 | 9.47 | (−) |
| 113 | EPA | PGD_3_ | PGF_2α_-d_4_ | 349.2 | 269.2 | 9.8 | (−) |
| 114 | EPA | LXA_5_ | PGE_2_-d_4_ | 349.2 | 115.1 | 10.47 | (−) |
| 115 | EPA | 17,18-DiHETE | LTB_4_-d_4_ | 335.2 | 247.2 | 13.94 | (−) |
| 116 | EPA | 14,15-DiHETE | LTB_4_-d_4_ | 335.2 | 207.2 | 14.31 | (−) |
| 117 | EPA | 5,6-DiHETE | LTB_4_-d_4_ | 335.2 | 145.1 | 14.84 | (−) |
| 118 | EPA | 18-HEPE | LTB_4_-d_4_ | 317.2 | 215.2 | 15.86 | (−) |
| 119 | EPA | 15-HEPE | 15-HETE-d_8_ | 317.2 | 219.2 | 16.2 | (−) |
| 120 | EPA | 12-HEPE | 15-HETE-d_8_ | 317.2 | 179.1 | 16.385 | (−) |
| 121 | EPA | 5-HEPE | 15-HETE-d_8_ | 317.2 | 115.1 | 16.45 | (−) |
| 122 | EPA | 15-HpEPE | 15-HETE-d_8_ | 333.2 | 111.1 | 16.56 | (−) |
| 123 | EPA | 12-HpEPE | 15-HETE-d_8_ | 333.2 | 271.2 | 16.67 | (−) |
| 124 | EPA | 5-HpEPE | 15-HETE-d_8_ | 333.2 | 173.1 | 16.865 | (−) |
| 125 | EPA | 17,18-EpETE | 15-HETE-d_8_ | 317.2 | 255.2 | 16.96 | (−) |
| 126 | DHA | DHA | DHA-d_5_ | 327.2 | 283.2 | 20.16 | (−) |
| 127 | DHA | Resolvin D_2_ | PGD_2_-d_4_ | 375.2 | 175.1 | 11.355 | (−) |
| 128 | DHA | Resolvin D_1_ | PGD_2_-d_4_ | 375.2 | 141.1 | 11.96 | (−) |
| 129 | DHA | Maresin1 | LTB_4_-d_4_ | 359.2 | 177.1 | 14.09 | (−) |
| 130 | DHA | 10,17-DiHDoHE | LTB_4_-d_4_ | 359.2 | 153.1 | 14.17 | (−) |
| 131 | DHA | 7,17-hydroxy-DPA | LTB_4_-d_4_ | 361.2 | 143.1 | 14.41 | (−) |
| 132 | DHA | 20-HDoHE | 15-HETE-d_8_ | 343.2 | 241.2 | 16.77 | (−) |
| 133 | DHA | 16-HDoHE | 12-HETE-d_8_ | 343.2 | 233.2 | 17.05 | (−) |
| 134 | DHA | 17-HDoHE | 12-HETE-d_8_ | 343.2 | 245.2 | 17.08 | (−) |
| 135 | DHA | 13-HDoHE | 12-HETE-d_8_ | 343.2 | 193.1 | 17.15 | (−) |
| 136 | DHA | 10-HDoHE | 12-HETE-d_8_ | 343.2 | 153.1 | 17.2 | (−) |
| 137 | DHA | 14-HDoHE | 12-HETE-d_8_ | 343.2 | 205.2 | 17.24 | (−) |
| 138 | DHA | 11-HDoHE | 5-HETE-d_8_ | 343.2 | 149.1 | 17.31 | (−) |
| 139 | DHA | 7-HDoHE | 5-HETE-d_8_ | 343.2 | 141.1 | 17.31 | (−) |
| 140 | DHA | 8-HDoHE | 5-HETE-d_8_ | 343.2 | 109.1 | 17.37 | (−) |
| 141 | DHA | 4-HDoHE | 5-HETE-d_8_ | 343.2 | 101.1 | 17.67 | (−) |
| 142 | IS | tetranor-PGEM-d_6_ |  | 333.2 | 315.2 | 2.99 | (−) |
| 143 | IS | 6-keto-PGF_1α_-d_4_ |  | 373.2 | 249.1 | 7.64 | (−) |
| 144 | IS | TXB_2_-d_4_ |  | 373.2 | 199.1 | 9.445 | (−) |
| 145 | IS | PGF_2α_-d_4_ |  | 357.2 | 197.2 | 10.25 | (−) |
| 146 | IS | PGE_2_-d_4_ |  | 355.2 | 275.2 | 10.67 | (−) |
| 147 | IS | PGD_2_-d_4_ |  | 355.2 | 275.2 | 11.08 | (−) |
| 148 | IS | LTC_4_-d_5_ |  | 631.4 | 308.2 | 12.6 | (+) |
| 149 | IS | LTB_4_-d_4_ |  | 339.2 | 197.1 | 14.095 | (−) |
| 150 | IS | 15-HETE-d_8_ |  | 327.2 | 226.2 | 16.86 | (−) |
| 151 | IS | 12-HETE-d_8_ |  | 327.2 | 184.3 | 17.215 | (−) |
| 152 | IS | 5-HETE-d_8_ |  | 327.2 | 116.1 | 17.32 | (−) |
| 153 | IS | OEA-d_4_ |  | 330.2 | 66.1 | 19.11 | (+) |
| 154 | IS | EPA-d_5_ |  | 306.2 | 262.2 | 19.4 | (−) |
| 155 | IS | DHA-d_5_ |  | 332.2 | 288.2 | 20.13 | (−) |
| 156 | IS | AA-d_8_ |  | 311.2 | 311.2 | 20.26 | (−) |
