# Supplementary material for: The profile of lipid metabolites in urine of marmoset wasting syndrome
Source: PLoS One. 2020 Jun 23;15(6):e0234634. doi: 10.1371/journal.pone.0234634 (PMC7310677; doi:10.1371/journal.pone.0234634)
Supplement: Attachment — Submitted filename: PONE-D-20-02190_reviewerFC.pdf [file pone.0234634.s003.pdf]

PLOS ONE

The production profile of lipid metabolites in urine of marmoset with wasting syndrome

--Manuscript Draft--

**Manuscript Number:**	PONE-D-20-02190
**Article Type:**	Research Article
**Full Title:**	The production profile of lipid metabolites in urine of marmoset with wasting syndrome
**Short Title:**	Urinary lipid profiling of the marmoset wasting syndrome
**Corresponding Author:**	Takahisa Murata, D.V.M., Ph.D.<br>The University of Tokyo<br>Tokyo, JAPAN
**Keywords:**	Marmoset, Wasting syndrome, Urine, lipid metabolite, Inflammatory
**Abstract:**	Marmoset wasting syndrome (MWS) is clinically characterized by progressive weight loss. Although MWS morbidity and mortality in captive marmosets are relatively high, its causes remain unknown. Lipid mediators are bioactive metabolites which are produced from polyunsaturated fatty acids, such as arachidonic acid (AA) and eicosapentaenoic acid (EPA). These lipid metabolites regulate a wide range of inflammatory responses and they are excreted into the urine. As urinary lipid profiles reflect systemic inflammatory conditions, we comprehensively measured the levels of 141 types of lipid metabolites in the urines obtained from healthy common marmoset (Callithrix jacchus) (N = 7) to those with MWS (N = 7). We found that 41 types of metabolites were detected in all urine samples of common marmoset. Among them, AA-derived metabolites accounted for 63% (26 types) of the total. Notably, the levels of AA-derived prostaglandin E_2, $F_{2\alpha}$, a metabolite of thromboxane A_2 and F_2-isoprostanes significantly increased in the urines of MWS. Our findings may help to understand the mechanisms and/or timing of onset and progression of MWS.
**Order of Authors:**	Arisa Yamazaki
	Tatsuro Nakamura
	Takako Miyabe-Nishiwaki
	Akihiro Hirata
	Rikako Inoue
	Koji Kobayashi
	Yusuke Miyazaki
	Yuta Hamasaki
	Akiyo Ishigami
	Nanae Nagata
	Akihisa Kaneko
	Makoto Koizumi
	Satoshi Maeda
	Rie Nishii
	Hiroki Ohta
	Hirotaka James Okano
	Takahisa Murata, D.V.M., Ph.D.
**Additional Information:**	
**Question**	**Response**
**Financial Disclosure**	This work was supported by the Cooperative Research Program of Primate Research

Powered by Editorial Manager® and ProduXion Manager® from Aries Systems Corporation

Enter a financial disclosure statement that describes the sources of funding for the work included in this submission. Review the submission guidelines for detailed requirements. View published research articles from *PLOS ONE* for specific examples.<br><br>This statement is required for submission and **will appear in the published article** if the submission is accepted. Please make sure it is accurate.<br><br>**Unfunded studies**<br>Enter: *The author(s) received no specific funding for this work.*<br><br>**Funded studies**<br>Enter a statement with the following details:<br>• Initials of the authors who received each award<br>• Grant numbers awarded to each author<br>• The full name of each funder<br>• URL of each funder website<br>• Did the sponsors or funders play any role in the study design, data collection and analysis, decision to publish, or preparation of the manuscript?<br>• **NO** - Include this sentence at the end of your statement: *The funders had no role in study design, data collection and analysis, decision to publish, or preparation of the manuscript.*<br>• **YES** - Specify the role(s) played.<br><br>* typeset	Institute, Kyoto University, the Japan Society for the Promotion of Science, the Takeda Science Foundation, the Nipponham Foundation, Hoyu Science Foundation, and the Futaba Foundation to T.M..
**Competing Interests**<br><br>Use the instructions below to enter a competing interest statement for this submission. On behalf of all authors, disclose any competing interests that could be perceived to bias this work—acknowledging all financial support and any other relevant financial or non-financial competing interests.<br><br>This statement **will appear in the published article** if the submission is accepted. Please make sure it is accurate. View published research articles	The authors have declared that no competing interests exist.

from *PLOS ONE* for specific examples.<br><br>**NO authors have competing interests**<br><br>Enter: *The authors have declared that no competing interests exist.*<br><br>**Authors with competing interests**<br><br>Enter competing interest details beginning with this statement:<br><br>*I have read the journal's policy and the authors of this manuscript have the following competing interests: [insert competing interests here]*<br><br>* typeset	
**Ethics Statement**<br><br>Enter an ethics statement for this submission. This statement is required if the study involved:<br><br>• Human participants<br>• Human specimens or tissue<br>• Vertebrate animals or cephalopods<br>• Vertebrate embryos or tissues<br>• Field research<br><br>Write "N/A" if the submission does not require an ethics statement.<br><br>General guidance is provided below. Consult the submission guidelines for detailed instructions. **Make sure that all information entered here is included in the Methods section of the manuscript.**	All procedures used in this study followed the Guidelines for the Care and Use of Nonhuman Primates, provided by Primate Research Institute, Kyoto University (KUPRI). The protocol for urinary sampling was approved by Institutional Animal Care and Use Committee in KUPRI (approval number: 2018-079).

Powered by Editorial Manager® and ProduXion Manager® from Aries Systems Corporation

**Format for specific study types**<br><br>**Human Subject Research (involving human participants and/or tissue)**<br>• Give the name of the institutional review board or ethics committee that approved the study<br>• Include the approval number and/or a statement indicating approval of this research<br>• Indicate the form of consent obtained (written/oral) or the reason that consent was not obtained (e.g. the data were analyzed anonymously)<br><br>**Animal Research (involving vertebrate animals, embryos or tissues)**<br>• Provide the name of the Institutional Animal Care and Use Committee (IACUC) or other relevant ethics board that reviewed the study protocol, and indicate whether they approved this research or granted a formal waiver of ethical approval<br>• Include an approval number if one was obtained<br>• If the study involved *non-human primates*, add *additional details* about animal welfare and steps taken to ameliorate suffering<br>• If anesthesia, euthanasia, or any kind of animal sacrifice is part of the study, include briefly which substances and/or methods were applied<br><br>**Field Research**<br><br>Include the following details if this study involves the collection of plant, animal, or other materials from a natural setting:<br>• Field permit number<br>• Name of the institution or relevant body that granted permission	
**Data Availability**<br><br>Authors are required to make all data underlying the findings described fully available, without restriction, and from the time of publication. PLOS allows rare exceptions to address legal and ethical concerns. See the PLOS Data Policy and FAQ for detailed information.	Yes - all data are fully available without restriction

A Data Availability Statement describing where the data can be found is required at submission. Your answers to this question constitute the Data Availability Statement and **will be published in the article**, if accepted.<br><br>**Important:** Stating 'data available on request from the author' is not sufficient. If your data are only available upon request, select 'No' for the first question and explain your exceptional situation in the text box.<br><br>Do the authors confirm that all data underlying the findings described in their manuscript are fully available without restriction?	
**Describe where the data may be found in full sentences. If you are copying our sample text, replace any instances of XXX with the appropriate details.**<br><br>• If the data are **held or will be held in a public repository**, include URLs, accession numbers or DOIs. If this information will only be available after acceptance, indicate this by ticking the box below. For example: *All XXX files are available from the XXX database (accession number(s) XXX, XXX.).*<br>• If the data are all contained **within the manuscript and/or Supporting Information files**, enter the following: *All relevant data are within the manuscript and its Supporting Information files.*<br>• If neither of these applies but you are able to provide **details of access elsewhere**, with or without limitations, please do so. For example:<br><br>*Data cannot be shared publicly because of [XXX]. Data are available from the XXX Institutional Data Access / Ethics Committee (contact via XXX) for researchers who meet the criteria for access to confidential data.*<br><br>*The data underlying the results presented in the study are available from (include the name of the third party*	All relevant data are within the manuscript and its Supporting Information files.

and contact information or URL).<br>• This text is appropriate if the data are owned by a third party and authors do not have permission to share the data.<br><br>* typeset	
Additional data availability information:	

Powered by Editorial Manager® and ProduXion Manager® from Aries Systems Corporation

Manuscript
Click here to access/download;Manuscript;200203 MWS_manuscript_plos resubmit_ver.docx

The production profile of lipid metabolites in urine of marmoset wasting syndrome

Urinary lipid profiling of the marmoset wasting syndrome

Arisa Yamazaki[1¶], Tatsuro Nakamura[1¶], Takako Miyabe-Nishiwaki[2], Akihiro Hirata[3], Rikako Inoue[1], Koji Kobayashi[1], Yusuke Miyazaki[1], Yuta Hamasaki[1], Akiyo Ishigami[2], Nanae Nagata[1], Akihisa Kaneko[2], Makoto Koizumi[4], Satoshi Maeda[5], Rie Nishii[5], Hiroki Ohta[6], Hirotaka James Okano[6], Takahisa Murata[1]*

[1] Department of Animal Radiology, Graduate School of Agriculture and Life Sciences, The University of Tokyo, Tokyo, Japan.

[2] Department of Primate Research Institute, Kyoto University, Aichi, Japan.

[3] Department of Animal Experiment, Life Science Research Center, Gifu University, Gifu, Japan.

[4] Department of Laboratory Animal Facilities, The Jikei University School of Medicine, Tokyo, Japan.

[5] Marmoset Breeding Facility, CLEA Japan, Inc, Gifu, Japan

[6] Department of Regenerative Medicine, The Jikei University School of Medicine, Tokyo, Japan.

* Corresponding author

E-mail: amurata@mail.ecc.u-tokyo.ac.jp (TM)

¶ These authors contributed equally to this work.

Abstract

Marmoset wasting syndrome (MWS) is clinically characterized by progressive weight loss. Although MWS morbidity and mortality in captive marmosets are relatively high, its causes remain unknown. Lipid mediators are bioactive metabolites which are produced from polyunsaturated fatty acids, such as arachidonic acid (AA) and eicosapentaenoic acid (EPA). These lipid metabolites regulate a wide range of inflammatory responses and they are excreted into the urine. As urinary lipid profiles reflect systemic inflammatory conditions, we comprehensively measured the levels of 141 types of lipid metabolites in the urines obtained from healthy common marmoset (*Callithrix jacchus*) (N = 7) to those with MWS (N = 7). We found that 41 types of metabolites were detected in all urine samples of common marmoset. Among them, AA-derived metabolites accounted for 63% (26 types) of the total. Notably, the levels of AA-derived prostaglandin (PG) E_2, $PGF_{2\alpha}$, a metabolite of thromboxane (TX) A_2, TXB_2 and F_2-isoprostanes significantly increased in the urines of MWS. Our findings may help to understand the mechanisms and/or timing of onset and progression of MWS.

Introduction

Marmoset wasting syndrome (MWS) is one of the leading causes of morbidity and mortality in captive marmosets [1]. The common clinical signs of MWS are chronic weight loss, diarrhea, and anemia. Although several different lesions have been pathologically found in marmosets with clinical signs of MWS [2, 3], chronic colitis is considered one of the most important contributing factors for the development MWS [4-6]. Steroid, tranexamic acid, and glucocorticoid are often used to treat symptoms, however, there is no reliable treatment for MWS [1, 7]. There is also no definitive diagnosis method for MWS.

Polyunsaturated free fatty acids (PUFAs) are divided into omega-6 (*n*-6) and omega-3 (*n*-3) groups based on double bond positions. The *n*-6 PUFAs include linoleic acid (LA), dihomo-γ-linolenic acid (DGLA), and arachidonic acid (AA), whereas *n*-3 PUFAs include α-linoleic acid (ALA), eicosapentaenoic acid (EPA), and docosahexaenoic acid (DHA). Three types of oxygenase, cyclooxygenase (COX), lipoxygenase (LOX), and cytochrome P450 (CYP) epoxygenase oxidize PUFAs and convert to lipid mediators and/or their related metabolites [8].

Lipid mediators are bioactive molecules and have identified hundreds of different types in the mammalian body. It has been demonstrated that lipid mediators positively and/or negatively regulate inflammatory responses [9]. For example, AA-derived COX metabolite prostaglandin E_2

(PGE_2) induces arterial dilation and vascular hyper-permeability resulting in edema, which is a classic sign of inflammation [10]. Another AA-derived pro-inflammatory mediator leukotriene B_4 induces chemotaxis and the production of pro-inflammatory cytokines in neutrophils [11, 12]. In contrast, the EPA-derived LOX metabolite resolvin exerts anti-inflammatory properties by inhibiting the recruitment of leukocytes/neutrophils and/or the production of pro-inflammatory cytokines [13].

Lipid mediators and their related metabolites are present in the urine and their levels change according to the host inflammatory condition. Indeed, some specific lipid metabolites positively correlate with disease activity score in human rheumatoid arthritis [14]. In addition, our previous studies demonstrate that a major urinary metabolite of prostaglandin D_2 (PGD_2), tetranor-PGDM, positively correlates with the severity of food allergies in mice and humans [15]. Thus, the profiles of urinary lipid metabolites can be convenient indices of various types of diseases.

On the basis of these backgrounds, we performed an LC-MS-based comprehensive analysis of lipid metabolites in the urines of marmosets suffering from MWS to understand pathophysiological features and to provide some insight for the development of a diagnostic methods.

Materials and Methods

Humane care guidelines

All procedures used in this study followed the Guidelines for the Care and Use of Nonhuman Primates, provided by Primate Research Institute, Kyoto University (KUPRI). The protocol for urinary sampling was approved by Institutional Animal Care and Use Committee in KUPRI (approval number: 2018-079). We did not anesthetize or euthanize animals for the purpose of this study. No specific animal research protocol was drafted for this in the Jikei University School of Medicine (Jikei) and Marmoset Breeding Facility, CLEA Japan, Inc. study as only clinical samples were provided to the study.

Rearing conditions of marmosets

The marmosets were housed in family cages (W700 x D700 x H1500 mm or W910 x D700 x H1600 mm in KUPRI, W800 mm, D650 mm, H1500 mm in Jikei), paired cages (W1200 x D600 x H1000 mm in KUPRI, W800 mm, D650 mm, H720 mm in Jikei.) or individual/intensive care cages (W600 x D600 x H1000 mm in KUPRI, W400 mm, D650 mm, H720 mm in Jikei, W390 mm, D550 mm, H700 mm in CLEA). The room temperature is kept at 28 ± 5°C. Water was available ad libitum.

They were fed on 50 ml (20-30 g) pellet (SPS, Oriental Yeast Co. ltd., Tokyo, Japan) twice a day supplemented with apple and quail's egg three times a week, banana twice a week and occasional mealworm (the larva of Tenebrionidae) in KUPRI. In Jikei, they were fed on 40 g pellet (LabDiet, PMI Nutrition International LLC, U.S.A) twice a day supplemented with honey, Vitamin C and Vitamin D. In CLEA, they were fed on 40-50 g CMS-1M（CLEA japan, Inc., Tokyo, Japan）soaked in water and supplemented with Vitamin C and D once a day, and boiled eggs twice a week. In all institutions, environmental enrichment such as gum feeders, wooden toys, climbing structures and swings are provided depending on the housing condition. The animal care staffs observe the health and well-being of the animals daily using criteria such as fecal condition, appetite, hair condition and movement and if they suspect any problems, they would consult with veterinarians. After the study, control marmosets were used in different projects and MWS marmosets underwent further treatment.

Urine samples

The urine samples were collected from common marmosets (*Callithrix jacchus*) diagnosed as healthy (N = 7) in primary care or MWS (N = 7). The information of each marmoset is shown in the Table 1. In general, healthy adult marmosets weigh around 350 g in captivity [16]. In this study, the body weight of each healthy marmoset exceeded 300 g (except for No.4, which was 1 year old

and still growing), however, that of MWS were around or less than 300 g at urine collection. Frequent and recurrent diarrhea, and weight loss were observed in MWS. Marmosets with MWS had a history of medication, such as ursodeoxycholic acid and pancrelipase, but which had been taken approximately 1 month before urine collection. The urines were collected from a tray under the cage after confirming that no feces were contaminated.

Table 1. Body weight and clinical signs in individual animals.

	**No.**	**Institution**	**Gender**	**Age (year)**	**BW (g)**	**Clinical signs**
**Healthy**	1	KUPRI	male	4	354	None
	2	KUPRI	male	6	378	None
	3	Jikei	male	9	320	None
	4	CLEA	female	1	290	None
	5	CLEA	male	2	370	None
	6	CLEA	male	1	340	None
	7	CLEA	male	2	310	None
**MWS**	1	KUPRI	male	6	280	frequent, recurring diarrhea, unexplained weight loss
	2	Jikei	male	4	193	unexplained weight loss
	3	CLEA	male	3	245	recurring diarrhea

	4	CLEA	male	9	230	recurring diarrhea
	5	CLEA	male	6	305	recurring diarrhea
	6	CLEA	female	7	290	recurring diarrhea
	7	CLEA	female	5	210	recurring diarrhea

KUPRI, Kyoto University Primate Research Institute; Jikei, The Jikei University School of Medicine; CLEA, Marmoset Breeding Facility, CLEA Japan, Inc.

Sample preparation

After the urine samples were centrifuged at 20,000 x *g* for 5 min, aliquot of 200 µl urine was mixed with 350 µl of 0.1% formic acid water and 50 µl of the internal standard solution. The mixed solutions were applied to solid phase extraction cartridge (OASIS µElution plate, Waters, Massachusetts, USA) preconditioned with 200 µl methanol and distilled water (DW). After washing with 200 µl DW and 200 µl hexane, lipids fractions were eluted with 100 µl methanol.

LC-MS-based comprehensive analysis of lipid metabolites

The 5 µl sample solution was injected to high performance liquid chromatography (Nexera 2, Shimadzu, Kyoto, Japan) equipped with mass spectrometer (LCMS-8060, Shimadzu, Kyoto, Japan). 138 types of metabolites, 3 types of PUFAs and 15 types of internal standards were

measured and analyzed by using LC/MSMS Method Package for Lipid Mediators version 2 with LabSolutions software (Shimadzu, Kyoto, Japan) as manufacturers instruction (S1 Table). Each metabolite was identified by retention time and selected reaction monitoring ion transition (S1 Table). The change of each metabolite level between healthy and MWS urines was examined by comparing the peak area ratio calculated as following formula; The peak area of each metabolite / the peak area of internal standards. Each value was further corrected by the concentration of creatinine measured by LabAssay™ Creatinine (Wako, Osaka, Japan).

Statistical analysis

All data are shown as mean ± SEM. The statistical difference was determined by Mann-Whitney U test for two-group comparison. Statistically significance was determined when p-value is less than 0.05.

Results

Detected lipid metabolites in the urines of MWS

We comprehensively measured 141 types of lipid metabolites including 3 types of PUFAs (AA, EPA and DHA) using urine from healthy (N = 7) and MWS (N = 7) marmosets (Table 1 and S1

Table). To unveil the inflammatory features of MWS, we focused and analyzed the metabolites that were constantly detected in all MWS individuals. Under these criteria, we detected 3 types of PUFAs (Fig 1) and 38 types of metabolites in total (Figs 2-4). In the detected 38 types of metabolites, *n*-6 PUFAs-derived metabolites accounted for 83% (34/38 types), on the other hand, the rest of metabolites were consisted of *n*-3 PUFAs-derived metabolites (7/38 types). Furthermore, AA-derived metabolites accounted for 65% (25/38 types) of total detected metabolites. The amount of AA tended to decrease but that EPA or DHA did not change in MWS urines compared to healthy urines (Fig 1). These results suggest that *n*-6 fatty acids, especially AA, are mainly consumed in MWS marmosets.

Fig 1. The urinary level of PUFAs.

The urinary levels of arachidonic acid (AA), eicosapentaenoic acid (EPA) and docosahexaenoic acid (DHA) in heathy (N = 7) and MWS (N = 7).

Fig 2. The urinary levels of AA-derived enzymic-oxidative metabolites.

The urinary levels of arachidonic acid (AA)-derived catalyzed metabolites in heathy (N = 7) and MWS (N = 7). di. dihydro; te, tetranor; COX, Cyclooxygenase; CYP, CYP450 epoxygenase; LOX,

lipoxygenase. *p<0.05, **p<0.01 compared to healthy urines.

Fig 3. The urinary levels of AA-derived isoprostanes.

The urinary levels of arachidonic acid (AA)-derived isoprostanes produced by non-enzymatic oxidation in healthy (N = 7) and MWS (N = 7). **p<0.01 compared to healthy urines.

Fig 4. The urinary levels of lipid metabolites derived from PUFAs except AA.

The urinary levels of (A) linoleic acid (LA), (B) dihomo-γ-linolenic acid (DGLA), (C) α-linoleic acid (ALA), (D) eicosapentaenoic acid (EPA) and (E) docosahexaenoic acid (DHA)-derived metabolite in healthy (N = 7) and MWS (N = 7). *p<0.05 compared to healthy urines. LOX, lipoxygenase; COX, cyclooxygenase; CYP, cytochrome P450.

Metabolic pathways of detected lipid metabolites

The metabolic pathways of all detected metabolites were drawn in Fig 2, 3 and 4. To determine which lipid mediators are critically involved in the development of MWS, we focused on the lipid metabolites that significantly increased in the urines of MWS than that of healthy. As shown

in Fig 2, the major AA metabolites, PGE_2, $PGF_{2\alpha}$ and its metabolite of 13, 14-dihydro-15-keto-tetranor-$PGF_{2\alpha}$ and a metabolite of TXA_2, TXB_2 were detected at ~~significant~~ higher levels in the urines of MWS than that of healthy. These results suggest that those three types of mediators may play important roles in the development and progression of MWS (Fig 2).

Isoprostanes are non-enzymatic oxidative product of PUFAs (Isoprostanes: an overview and putative roles in pulmonary pathophysiology). F_2-isprostanes (F_2-isoPs) are prostaglandin F_2-liked isoprostanes generated from AA. In this study, 5 types of F_2-isoPs were detected and 4 types of that significantly increased in MWS urines (Fig 3). Several studies reported that the amount of F_2-isoPs were elevated under oxidative stress upon inflammation [17, 18]. Thus, detection of these F_2-isoPs in urine may evidence the presence of inflammation associated oxidative stress in MWS.

In addition, DGLA-derived F_1-isoprostane (F_1-IsoP), 8-iso-PGE_1, also significantly increased in the urines of MWS (Fig 4). The other PUFAs-derived urinary metabolites did not change between healthy and MWS group (Fig 4). It is reported that F_1-IsoPs also produced by oxidative stress, however, the usefulness of urinary F_1-IsoP as biomarker has not yet been reported. 8-iso-PGE_1 may be a unique metabolite increased in MWS urines.

Discussion

Several stimuli immediately activate appropriate oxidases, such as COX and LOX, and lead *de novo* synthesis of lipid mediators from *n*-6 and/or *n*-3 PUFAs. Generally, *n*-6 PUFA AA-derived lipid mediators accelerate inflammation pathways in both the acute and chronic phases. On the other hand, *n*-3 PUFAs EPA- and DHA-derived mediators accelerate the resolution of inflammation at the chronic phase [19, 20]. Thus, the number, types, and levels of lipid mediators are changed according to the severity and/or duration of inflammation. The urinary lipid profiling reflects these changes in host inflammatory condition as the dynamic balance between *n*-6 and *n*-3 PUFAs-derived metabolites. In the present study, *n*-6 PUFA, especially AA, -derived metabolites accounted for a majority of urinary lipid metabolites in MWS. These results may reflect that MWS marmosets may have persistent inflammation accompanied by AA consumption.

Even though PGE_2 and $PGF_{2\alpha}$ are highly metabolically unstable, these compounds were detected in the urines of marmoset. In addition to PGE_2 and $PGF_{2\alpha}$, primary metabolites of these PGs in plasma, 13,14-dihydro-15-keto derivatives, were increased in the urines of MWS [21]. Thus, a large amount of these PGs would be produced in the inflammatory lesions and excreted into the MWS urines. However, previous studies have shown that some of the urinary PGE_2 or $PGF_{2\alpha}$ are formed by free-radical catalyzed oxidation pathway (IsoP pathway) in the rodent and human [22, 23]. It cannot be ignored the possibility that urinary PGE_2 and/or $PGF_{2\alpha}$ were generated by IsoP pathway and the

increase their levels reflect a free radical production by oxidative stress.

Chronic enteritis is a major post-mortal pathological feature of MWS [4-6]. We have also found many cases with severe chronic enteritis in marmosets clinically diagnosed with MWS in the colony at the Primate Research Institute, Kyoto University. In the present study, we found that the amounts of PGE_2, $PGF_{2\alpha}$ and a metabolite or TXA_2 were markedly increased in the urines of MWS. Clinical studies have shown that PGE_2 levels are elevated in the urine of patients with intestinal inflammation [24]. Gene deficiency or pharmacological blocking of the PGE_2 receptor, EP4, ameliorated dextran sodium sulfate–induced colitis in mice [25]. Collins D et al. showed in an *in vitro* study that $PGF_{2\alpha}$ promoted chloride secretion in human colonic epithelial cells through a cAMP-mediated mechanism [26]. Further, TXA_2 up-regulates neutrophil elastase release and aggravate trinitrobenzene sulfonic acid-induced guinea-pig colitis [27]. Our findings and those reports suggest that PGE_2, $PGF_{2\alpha}$ and TXA_2 may play important roles in the MWS development and progression of MWS. Further investigations are needed to reveal the functional relevance of these mediators in MWS.

In this study, we found that F_2-IsoPs were increased in the MWS urine. It is well known that the levels of urinary F_2-IsoPs elevated in the presence of oxidative stress. Indeed, urinary levels of F_2-isoP is elevated in the patients with Chron’s diseases [28]. Considering that isoprostanes are chemically stable, urinary F_2-IsoPs may be urinary biomarker of MWS. In addition to F_2-IsoPs, we

also found the increased level of F_1-isoP, 8-iso $PGF_{1\square}$, which possibly be unique in MWS urines. To demonstrate the relevance of the urinary levels of F_2-isoPs and F_1-IsoP to the onset of MWS may be able to establish a new early diagnosis method for MWS.

The *n*-3 PUFAs are essential fatty acid that is identified as an anti-inflammatory modulator by inhibiting AA metabolism and/or producing mediators such as resolvin, lipoxin, and protectin [29]. The decreased levels of *n*-3 PUFAs were reported in patients with inflammatory bowel disease. In this study, only 5 types of *n*-3 PUFAs-derived metabolites were constantly detected in MWS urines. Additionally, the levels of *n*-3 PUFAs did not change in both groups. These results raised the possibility that *n*-3 PUFAs are not metabolized in MWS marmoset. Several reports have suggested that supplementation with *n*-3 PUFAs improved inflammation, including inflammatory bowel disease [30, 31]. The feeding of *n*-3 PUFAs containing food may be effective for the treatment of MWS.

To our knowledge, this is the first study assessing the urinary lipid profile of MWS. Our findings may aid in clarifying the pathogenic mechanism for the development of MWS and also help develop indicators and/or markers for the diagnosis of MWS.

Acknowledgements

This work was supported by the Cooperative Research Program of Primate Research Institute, Kyoto University, the Japan Society for the Promotion of Science, the Takeda Science Foundation, the Nipponham Foundation, Hoyu Science Foundation, and the Futaba Foundation.

References

1. Otovic P, Smith S, Hutchinson E. The use of glucocorticoids in marmoset wasting syndrome. J Med Primatol. 2015; 44(2): 53-9. pmid: 25614344
2. Brack M, Rothe H. Chronic Tubulointerstitial Nephritis and Wasting Disease in Mamosets (Callithrux jacchus). Vet Pathol. 1981; 18(Suppl 6): 45-54. pmid: 6806966
3. Sousa MB, Leão AC, Coutinho JF, de Oliveira Ramos AM. Histopathology findings in common marmosets (Callithrix jacchus Linnaeus, 1758) with chronic weight loss associated with bile tract obstruction by infestation with Platynosomum (Loos, 1907). Primates. 2008; 49(4): 283-7. pmid: 18841441
4. Chalifoux LV, Bronson RT, Escajadillo A, McKenna S. An Analysis of the Association of Gastroenteric Lesions with Chronic Wasting Syndrome of Marmosets. Vet Pathol Suppl.

1982; 19 Suppl 7: 141-62. pmid: 6153004

5. Logan AC, Khan KN. Clinical Pathologic Changes in Two Marmosets with Wasting Syndrome. Toxicol Pathol. 1996; 24(6): 707-9. pmid: 8994297

6. Nakashima E, Okano Y, Niimi K, Takahashi E. Detection of Calprotectin and Apoptotic Activity in the Colon of Marmosets with Chronic Diarrhea. J Vet Med Sci. 2013. 75(12): 1633-1636. pmid: 23884022

7. Yoshimoto T, Niimi K, Takahashi E. Tranexamic Acid and Supportive Measures to Treat Wasting Marmoset Syndrome. Comp Med. 2016; 1;66(6): 468-473. pmid: 28304250

8. Michalak A, Mosińska P, Fichna J. Polyunsaturated Fatty Acids and Their Derivatives: Therapeutic Value for Inflammatory, Functional Gastrointestinal Disorders, and Colorectal Cancer. Front Pharmacol. 2016; 7: 459. pmid: 27990120

9. Bennett M, Gilroy DW. Lipid Mediators in Inflammation. Microbiol Spectr. 2016; 4(6). pmid: 27837747

10. Funk CD. Prostaglandins and leukotriens: advances in eicosanoid biology. Science. 2001; 294(5548): 1871-5. pmid: 11729303

11. Busse WW. Leukotriens and inflammation. Am J Respir Crit Care Med. 1998; 157(6 Pt1): S210-3. pmid: 9620941

12. Miles EA, Calder PC. Influence of marine n-3 polyunsaturated fatty acids on immune function and a systematic review of their effects on clinical outcomes in rheumatoid arthritis. Br J Nutr. 2012; 107 Suppl 2: p. S171-84. pmid: 22591891

13. Schwanke RC, Marcon R, Bento AF, Calixto JB. EPA- and DHA-derived resolvins' actions in inflammatory bowel disease. Eur J Pharmacol. 2016; 785: 156-164. pmid: 26325092

14. Fu J, Schoeman JC, Harms AC, van Wietmarschen HA, Vreeken RJ, Berger R, et al. Metabolomics profiling of the free and total oxidised lipids in urine by LC-MS/MS: application in patients with rheumatoid arthritis. Anal Bioanal Chem. 2016; 408(23): 6307-19. pmid: 27405874

15. Maeda S, Nakamura T, Harada H, Tachibana Y, Aritake K, Shimosawa T, et al. Prostaglandin D2 metabolite in urine is an index of food allergy. Sci Rep. 2017; 7(1): 17687. pmid: 29247205

16. Araújo A, Arruda MF, Alencar AI, Albuquerque F, Nascimento MC, Yamamoto ME. Body Weight of Wild and Captive Common Marmosets (Callithrix jacchus). International Journal of Primatology. 2000; 21(2): 317–324

17. Chiabrando C, Valagussa A, Rivalta C, Durand T, Guy A, Zuccato E, et al. Identification and measurement of endogenous beta-oxidation metabolites of 8-epi-Prostaglandin

F2alpha. J Biol Chem. 1999; 15;274(3): 1313-9. pmid: 9880501

18. Morrow JD, Hill KE, Burk RF, Nammour TM, Badr KF, Roberts LJ 2nd. A series of prostaglandin F2-like compounds are produced in vivo in humans by a non-cyclooxygenase, free radical-catalyzed mechanism. Proc Natl Acad Sci U S A. 1990; 87(23): 9383-7. pmid: 2123555

19. Arita, M. Mediator lipidomics in acute inflammation and resolution. J Biochem. 2012; 152(4): 313-9. pmid: 22923733

20. Fredman G, Serhan CN. Specialized proresolving mediator targets for RvE1 and RvD1 in peripheral blood and mechanisms of resolution. Biochem J. 2011; 437(2): 185-97. pmid: 21711247

21. Samuelsson B, Goldyne M, Granström E, Hamberg M, Hammarström S. Malmsten C. Prostaglandins and thromboxanes. Annu Rev Biochem. 1978; 47: p. 997-1029. pmid: 209733

22. Gao L, Zackert WE, Hasford JJ, Danekis ME, Milne GL, Remmert C, et al. Formation of prostaglandins E2 and D2 via the isoprostane pathway: a mechanism for the generation of bioactive prostaglandins independent of cyclooxygenase. J Biol Chem. 2003; 278(31): p. 28479-89. pmid: 12746435

23. Yin H, Gao L, Tai HH, Murphey LJ, Porter NA, Morrow JD. Urinary prostaglandin F2alpha is generated from the isoprostane pathway and not the cyclooxygenase in humans. J Biol Chem. 2007; 282(1): p. 329-36. pmid: 17107953

24. Arai Y, Matsuura T, Matsuura M, Fujiwara M, Okayasu I, Ito S, et al. Prostaglandin E-Major Urinary Metabolite as a Biomarker for Inflammation in Ulcerative Colitis: Prostaglandins Revisited. Digestion. 2016; 93(1): 32-9. pmid: 26788915

25. Kabashima K, Saji T, Murata T, Nagamachi M, Matsuoka T, Segi E, et al. The prostaglandin receptor EP4 suppresses colitis, mucosal damage and CD4 cell activation in the gut. Journal of Clinical Investigation. 2002; 109(7): 883-893. pmid: 11927615

26. Collins D, Hogan AM, Skelly MM, Baird AW, Winter DC. Cyclic AMP-mediated chloride secretion is induced by prostaglandin F2alpha in human isolated colon. Br J Pharmacol. 2009; 158(7): 1771-6. pmid: 19889058

27. Hirota Y, Suzuki M, Katsube N. Thromboxane A2 Up-Regulates Neutrophil Elastase Release in Syrian Hamsters With Trinitrobenzene Sulfonic Acid-Induced Colitis. J Pharmacol Sci. 2005; 98(4): 430-8. pmid: 16079461

28. Cracowski JL, Bonaz B, Bessard G, Bessard J, Anglade C, Fournet J. Increased urinary F2-isoprostanes in patients with Crohn's disease. Am J Gastroenterol. 2002; 97(1): 99-103.

pmid: 11808977

29. Duvall MG, Levy BD. DHA- and EPA-derived resolvins, protectins, and maresins in airway inflammation. Eur J Pharmacol. 2016; 785: 144-155. pmid: 26546247

30. Prossomariti A, Scaioli E, Piazzi G, Fazio C, Bellanova M, Biagi E, et al. Short-term treatment with eicosapentaenoic acid improves inflammation and affects colonic differentiation markers and microbiota in patients with ulcerative colitis. Sci Rep. 2017; 7(1): 7458. pmid: 28785079

31. Calder PC. Omega-3 polyunsaturated fatty acids and inflammatory processes: nutrition or pharmacology? Br J Clin Pharmacol. 2013; 75(3): 645-62. pmid: 22765297

Supporting information

S1 Table. Measured 141 types of lipid metabolites and 15 types of internal standards.

PUFA, polyunsaturated free fatty acid; SRM, selected reaction monitoring; ALA, α-linoleic acid; LA, linoleic acid; DGLA, dihomo-γ-linolenic acid; AA, arachidonic acid; EPA, eicosapentaenoic acid; DHA, docosahexaenoic acid.

Figure 1

Click here to access/download;Figure;Fig 1.tif

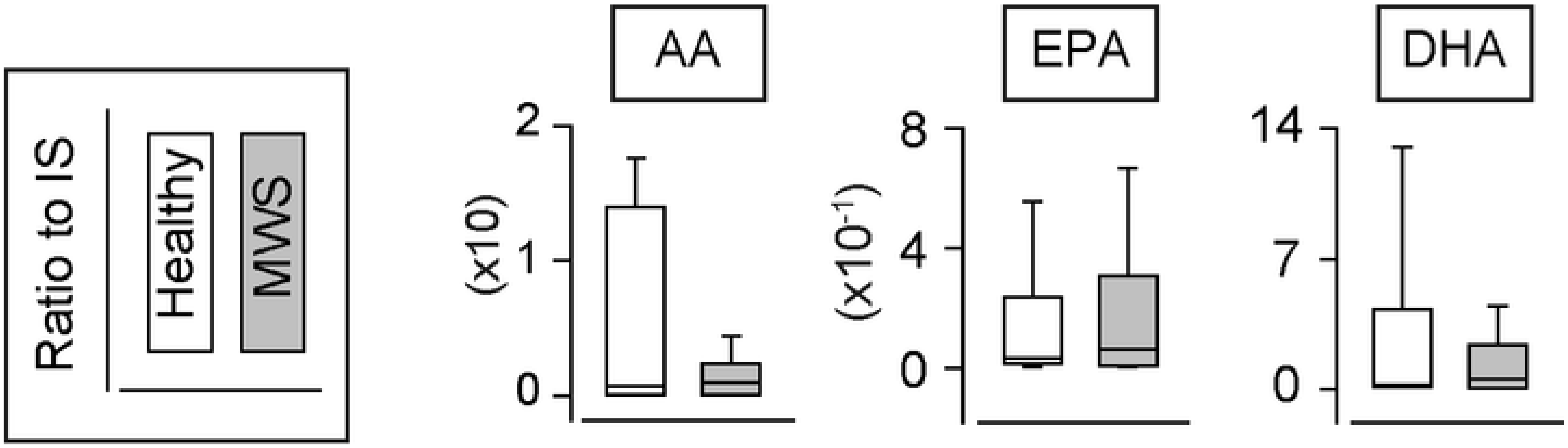

Figure 2 Click here to access/download;Figure;Fig 2.tif

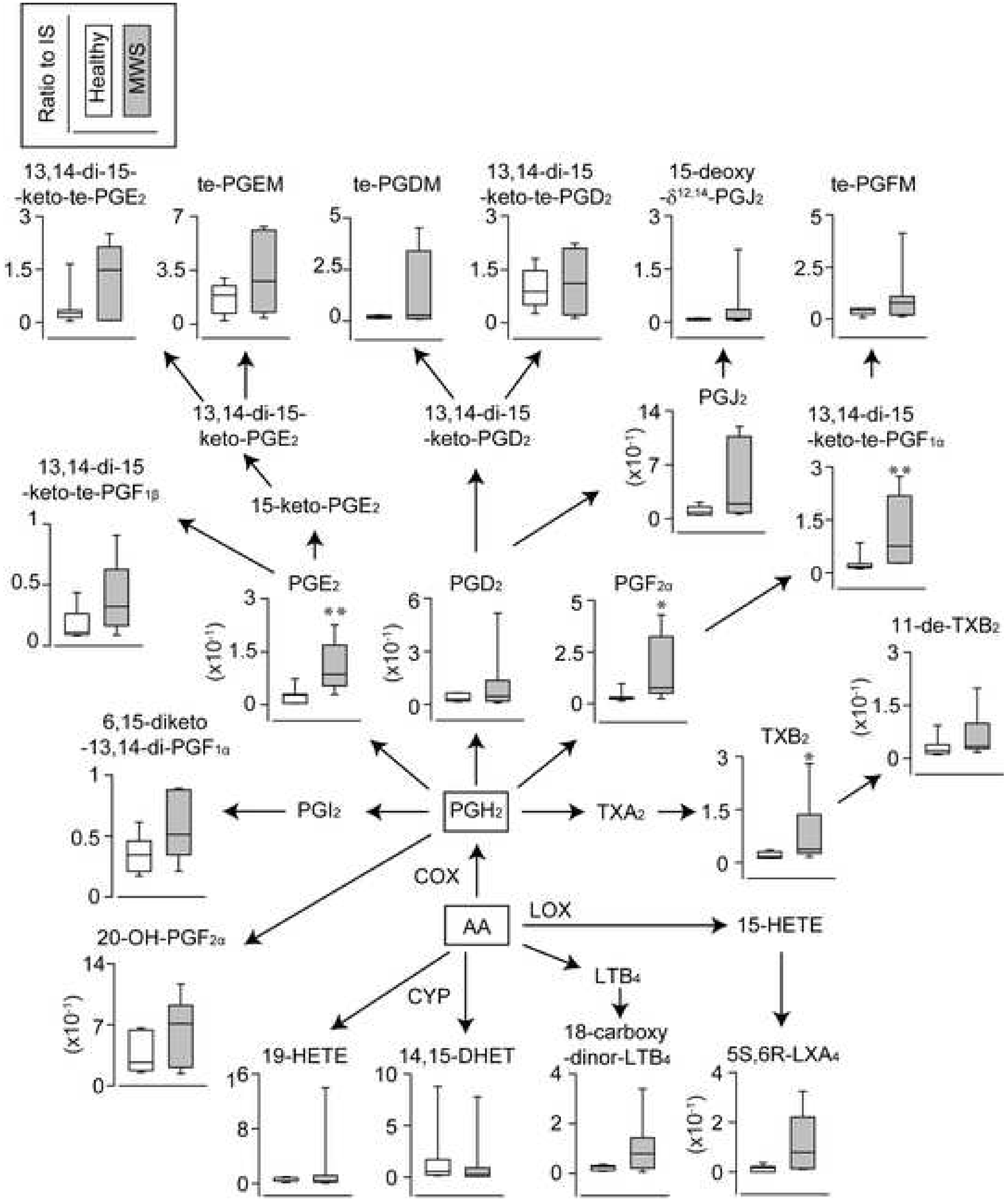

Figure 3

Click here to access/download;Figure;Fig 3.tif

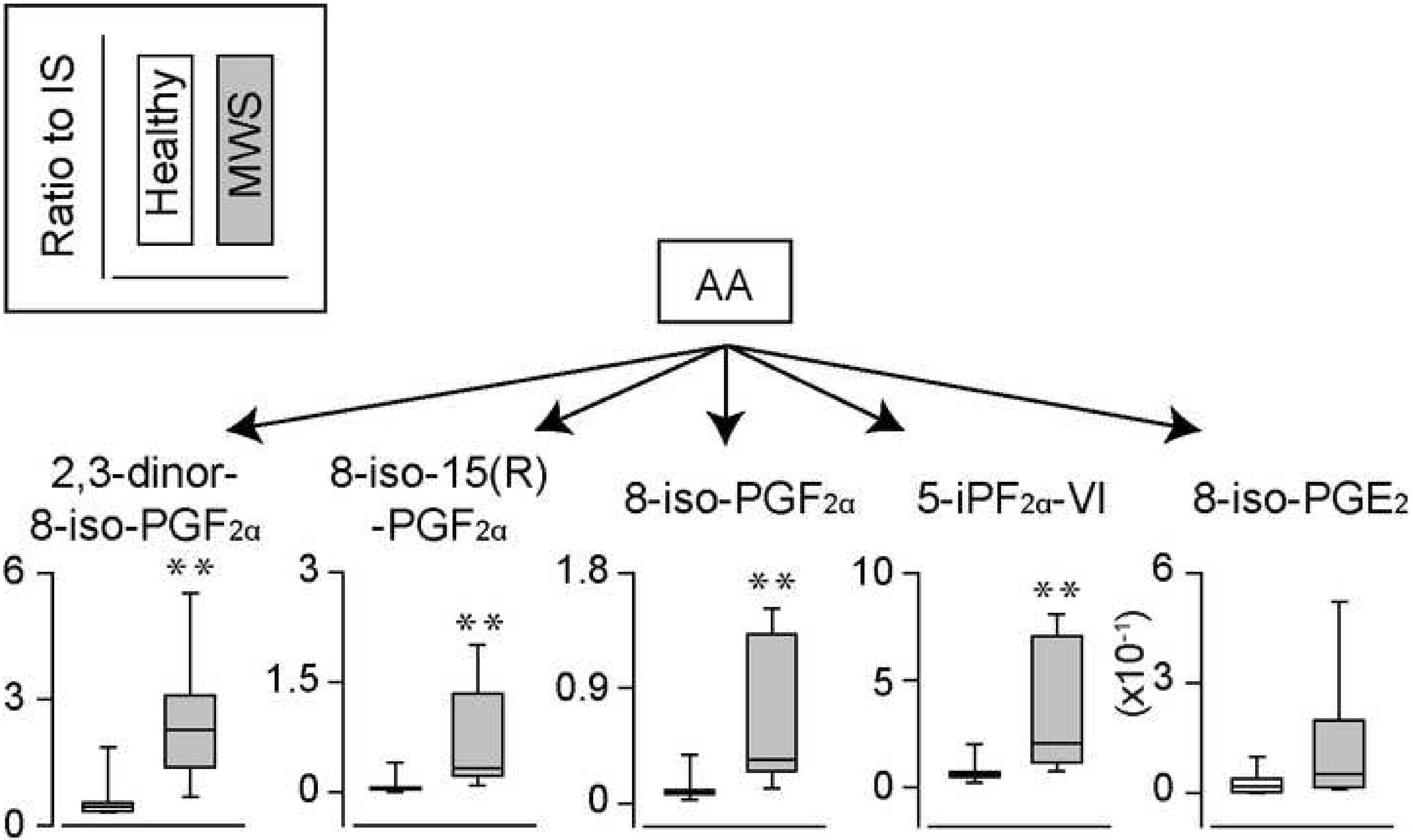

Figure 4 Click here to access/download;Figure;Fig 4.tif

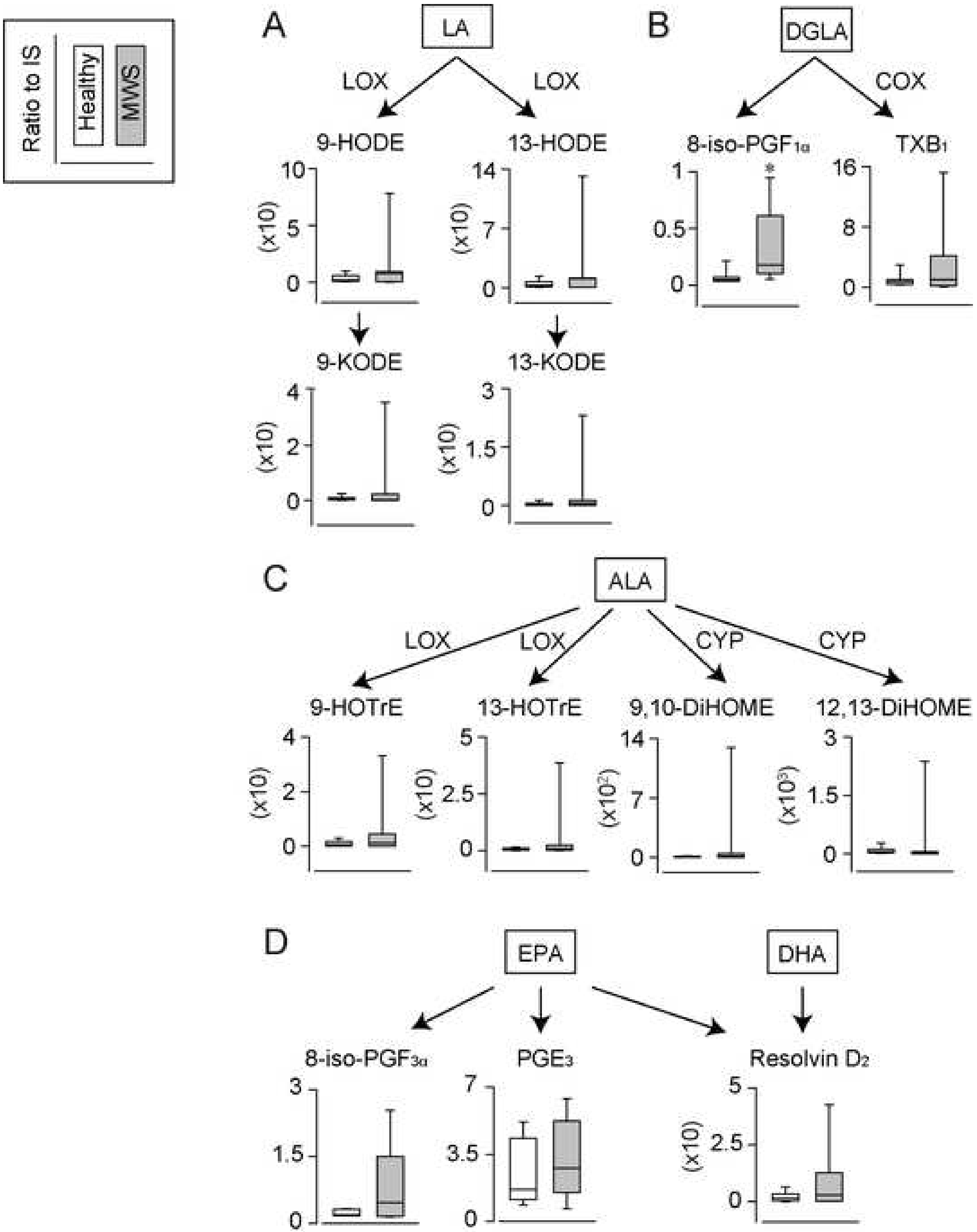

Click here to access/download
Supporting Information
200203 S1 Table.docx

“**The production profile of lipid metabolites in urine of marmoset wasting syndrome**”.

Reviewer's Responses to Questions

1. Is the manuscript technically sound, and do the data support the conclusions?

The manuscript must describe a technically sound piece of scientific research with data that supports the conclusions. Experiments must have been conducted rigorously, with appropriate controls, replication, and sample sizes. The conclusions must be drawn appropriately based on the data presented.

Reviewer #1: No

Reviewer #2: Partly

2. Has the statistical analysis been performed appropriately and rigorously?

Reviewer #1: No

Reviewer #2: No

3. Have the authors made all data underlying the findings in their manuscript fully available?

The PLOS Data policy requires authors to make all data underlying the findings described in their manuscript fully available without restriction, with rare exception (please refer to the Data Availability Statement in the manuscript PDF file). The data should be provided as part of the manuscript or its supporting information or deposited to a public repository. For example, in addition to summary statistics, the data points behind means, medians and variance measures should be available. If there are restrictions on publicly sharing data—e.g. participant privacy or use of data from a third party—those must be specified.

Reviewer #1: No

Reviewer #2: Yes

4. Is the manuscript presented in an intelligible fashion and written in standard English?

PLOS ONE does not copyedit accepted manuscripts, so the language in submitted articles must be clear, correct, and unambiguous. Any typographical or grammatical errors should be corrected at revision, so please note any specific errors here.

Reviewer #1: Yes

Reviewer #2: Yes

5. Review Comments to the Author

Please use the space provided to explain your answers to the questions above. You may also include additional comments for the author, including concerns about dual publication, research ethics, or publication ethics. (Please upload your review as an attachment if it exceeds 20,000 characters)

Reviewer #1: The paper describes analysis of PUFA metabolites from urine of Marmosets with the aim of identifying biomarkers for Marmoset wasting syndrome. The authors used LC-MS methods for the

characterization of the PUFA metabolome. Unfortunately, the manuscript is deficient on many fronts for it to be a valuable contribution.

1. The methods are extremely sketchy. There is no description of the internal standards used for the quantification of the lipid mediators.

Response: We added the list of the internal standard in S1 Table. In this study, we assessed the change of lipid profile by comparing the area ratio (calculated as target compound's peak area / the corresponding internal standard's peak area), but not by absolute concentration, between healthy and MWS urines. We added the description in Method section in line 123-127.

2. No details on how the lipids were identified (the supplementary data file only gives the abbreviations and names of the lipid metabolites).

Response: We identified each metabolite by retention time (RT) in LC and selected reaction monitoring (SRM) transition in MS/MS. The RT and the precursor and product m/z for SRM were added in S1 Table, and the description was added to Method section in line 123-127.

3. No mention of the actual levels of the metabolites measure (only fold-changes are reported).

Response: As mentioned in the response to comment 1, we assessed the change of lipid profile by comparing the area ratio (calculated as target compound's peak area / the corresponding internal standard's peak area), but not by the absolute concentration. This qualitative analysis enough to compare the production change of urinary lipid metabolites between healthy and MWS. We added the description in Method section in line 123-127.

4. Primary metabolites of PUFA by COX pathway are highly metabolically unstable to be detected in appreciable amounts even in plasma. No explanation of the detection of highly metabolically unstable lipids in urine (e.g. PGE_2, PGD_2, LTB_4, etc.) was offered.

Response: Thank you for suggestion. As the reviewer pointed out, even though PGE_2 and $PGF_{2\alpha}$ are highly metabolically unstable, these were detected in the urines of marmoset. In addition to PGE_2 and $PGF_{2\alpha}$, primary metabolites of these PGs in plasma, 13,14-dihydro-15-keto derivatives, were increased in the urines of MWS (1). Thus, a large amount of these PGs would be produced in the inflammatory lesions and excreted into the MWS urines. However, previous studies have shown that some of the urinary PGE_2 or PGF_{2a} are formed by free-radical catalyzed oxidation pathway (isoprostane pathway) in the rodent and human (2, 3). It cannot be ignored the possibility that urinary PGE_2 and/or PGF_{2a} were generated by isoprostanes pathway and the increase their levels reflect a free radical production by oxidative stress. We added the description in Discussion section in line 198-205.

1. Samuelsson B, Goldyne M, Granström E, Hamberg M, Hammarström S. Malmsten C. Prostaglandins and thromboxanes. Annu Rev Biochem. 1978; 47: p. 997-1029.
2. Gao L, Zackert WE, Hasford JJ, Danekis ME, Milne GL, Remmert C, et al. Formation of prostaglandins E2 and D2 via the isoprostane pathway: a mechanism for the generation of bioactive prostaglandins independent of cyclooxygenase. J Biol Chem. 2003; 278(31): p. 28479-89.

3. Yin H, Gao L, Tai HH, Murphey LJ, Porter NA, Morrow JD. Urinary prostaglandin F2alpha is generated from the isoprostane pathway and not the cyclooxygenase in humans. J Biol Chem. 2007; 282(1): p. 329-36.

5. The sample size of 3 for an in vivo study is extremely small to obtain statistically reliable data. Even under the best of conditions, LC-MS analysis of these lipids has 3-5% CV in technical replicates. What is the variability of the data between these samples? In fact, there is no mention of the p-values for any of these metabolites, to glean any reliability of the analysis or the data.
Response: We performed additional experiments with more 8 samples (healthy: 4 and MWS: 4) and the results were statistically analyzed. The statistical difference was determined by Mann-Whitney U test for two-group comparison. For this statistical analysis, the AA metabolites of PGE_2, $PGF_{2\alpha}$, TXB_2 and some isoprostanes were detected at significant higher levels in the urines of MWS than that of healthy. We added the description in Method section in line 130-133.

6. The urine samples were presumably collected from trays below the cages. One would assume that the trays would contain both urine and feces. It is not clear how the authors conclude that the metabolites detected were only urinary.
Response: We collected urinary samples after we visually confirmed that no feces were contaminated. We added the description in Method section in Table 1 and line 104-105.

Reviewer #2: Yamazaki et al reported a distinct profile of lipid metabolites in the urine of MWS. Authors identified increased 48 metabolites belonging to AA- and EPA-derived metabolites in the urine of MWS compared to healthy (N=3).
1. Please delete Section "Urine samples", its is written twice in manuscript.
Response: We deleted the word, thank you.

2. No statistical analysis was performed if identified metabolites are significantly altered or not. Number of animals in each group is of a BIG concern as 3 animals cannot be used for appropriate sample size for any in vivo studies. Authors are encouraged to increase the animal number in each group and perform statistical analysis. Without such data, this work has no impact.
Response: We increased 4 samples for each group and statistically analyzed 7 samples in each group. The statistical difference was determined by Mann-Whitney U test for two-group comparison. As a result, we found that AA metabolites of PGE_2, $PGF_{2\alpha}$, TXB_2 and some isoprostanes were detected at significant higher levels in the urines of MWS than that of healthy. We added the description in Method section in line 130-133.

3. One data point from MWS groups seems to be an outlier. Excluding this point, there is not difference in healthy and MWS groups. This point again pointing to increase the animal number in both groups for meaningful interpretation of data.

Response: As mentioned in the response to comment 1, we statistically analyzed with N = 7 data including the outlier sample, we found that some particular types of metabolites were detected at significant higher levels in the urines of MWS than that of healthy.

6. If you would like your identity to be revealed to the authors, please include your name here (optional).
Your name and review will not be published with the manuscript.
Reviewer #1: (No Response)
Reviewer #2: no
