# Supplementary material for: The profile of lipid metabolites in urine of marmoset wasting syndrome
Source: PLoS One. 2020 Jun 23;15(6):e0234634. doi: 10.1371/journal.pone.0234634 (PMC7310677; doi:10.1371/journal.pone.0234634)
Supplement: Attachment — Submitted filename: 20200124 MWS_response_reviewers.docx [file pone.0234634.s002.docx]

“**The production profile of lipid metabolites in urine of marmoset wasting syndrome**”.

Reviewer's Responses to Questions

1. Is the manuscript technically sound, and do the data support the conclusions?

The manuscript must describe a technically sound piece of scientific research with data that supports the conclusions. Experiments must have been conducted rigorously, with appropriate controls, replication, and sample sizes. The conclusions must be drawn appropriately based on the data presented.

Reviewer #1: No

Reviewer #2: Partly

2. Has the statistical analysis been performed appropriately and rigorously?

Reviewer #1: No

Reviewer #2: No

3. Have the authors made all data underlying the findings in their manuscript fully available?

The PLOS Data policy requires authors to make all data underlying the findings described in their manuscript fully available without restriction, with rare exception (please refer to the Data Availability Statement in the manuscript PDF file). The data should be provided as part of the manuscript or its supporting information or deposited to a public repository. For example, in addition to summary statistics, the data points behind means, medians and variance measures should be available. If there are restrictions on publicly sharing data—e.g. participant privacy or use of data from a third party—those must be specified.

Reviewer #1: No

Reviewer #2: Yes

4. Is the manuscript presented in an intelligible fashion and written in standard English?

PLOS ONE does not copyedit accepted manuscripts, so the language in submitted articles must be clear, correct, and unambiguous. Any typographical or grammatical errors should be corrected at revision, so please note any specific errors here.

Reviewer #1: Yes

Reviewer #2: Yes

5. Review Comments to the Author

Please use the space provided to explain your answers to the questions above. You may also include additional comments for the author, including concerns about dual publication, research ethics, or publication ethics. (Please upload your review as an attachment if it exceeds 20,000 characters)

**Reviewer #1:** The paper describes analysis of PUFA metabolites from urine of Marmosets with the aim of identifying biomarkers for Marmoset wasting syndrome. The authors used LC-MS methods for the characterization of the PUFA metabolome. Unfortunately, the manuscript is deficient on many fronts for it to be a valuable contribution.

1. The methods are extremely sketchy. There is no description of the internal standards used for the quantification of the lipid mediators.

**Response:** We added the list of the internal standard in S1 Table. In this study, we assessed the change of lipid profile by comparing the area ratio (calculated as target compound's peak area / the corresponding internal standard's peak area), but not by absolute concentration, between healthy and MWS urines. We added the description in Method section in line 123-127.

2. No details on how the lipids were identified (the supplementary data file only gives the abbreviations and names of the lipid metabolites).

**Response:** We identified each metabolite by retention time (RT) in LC and selected reaction monitoring (SRM) transition in MS/MS. The RT and the precursor and product m/z for SRM were added in S1 Table, and the description was added to Method section in line 123-127.

3. No mention of the actual levels of the metabolites measure (only fold-changes are reported).

**Response:** As mentioned in the response to comment 1, we assessed the change of lipid profile by comparing the area ratio (calculated as target compound's peak area / the corresponding internal standard's peak area), but not by the absolute concentration. This qualitative analysis enough to compare the production change of urinary lipid metabolites between healthy and MWS. We added the description in Method section in line 123-127.

4. Primary metabolites of PUFA by COX pathway are highly metabolically unstable to be detected in appreciable amounts even in plasma. No explanation of the detection of highly metabolically unstable lipids in urine (e.g. PGE_2_, PGD_2_, LTB_4_, etc.) was offered.

**Response:** Thank you for suggestion. As the reviewer pointed out, even though PGE_2_ and PGF_2α_ are highly metabolically unstable, these were detected in the urines of marmoset. In addition to PGE_2_ and PGF_2α_, primary metabolites of these PGs in plasma, 13,14-dihydro-15-keto derivatives, were increased in the urines of MWS (1). Thus, a large amount of these PGs would be produced in the inflammatory lesions and excreted into the MWS urines. However, previous studies have shown that some of the urinary PGE_2_ or PGF_2a_ are formed by free-radical catalyzed oxidation pathway (isoprostane pathway) in the rodent and human (2, 3). It cannot be ignored the possibility that urinary PGE_2_ and/or PGF_2a_ were generated by isoprostanes pathway and the increase their levels reflect a free radical production by oxidative stress. We added the description in Discussion section in line 198-205.

1. Samuelsson B, Goldyne M, Granström E, Hamberg M, Hammarström S. Malmsten C. Prostaglandins and thromboxanes. Annu Rev Biochem. 1978; 47: p. 997-1029.

2. Gao L, Zackert WE, Hasford JJ, Danekis ME, Milne GL, Remmert C, et al. Formation of prostaglandins E2 and D2 via the isoprostane pathway: a mechanism for the generation of bioactive prostaglandins independent of cyclooxygenase. J Biol Chem. 2003; 278(31): p. 28479-89.

3. Yin H, Gao L, Tai HH, Murphey LJ, Porter NA, Morrow JD. Urinary prostaglandin F2alpha is generated from the isoprostane pathway and not the cyclooxygenase in humans. J Biol Chem. 2007; 282(1): p. 329-36.

5. The sample size of 3 for an in vivo study is extremely small to obtain statistically reliable data. Even under the best of conditions, LC-MS analysis of these lipids has 3-5% CV in technical replicates. What is the variability of the data between these samples? In fact, there is no mention of the p-values for any of these metabolites, to glean any reliability of the analysis or the data.

**Response:** We performed additional experiments with more 8 samples (healthy: 4 and MWS: 4) and the results were statistically analyzed. The statistical difference was determined by Mann-Whitney U test for two-group comparison. For this statistical analysis, the AA metabolites of PGE_2_, PGF_2α_, TXB_2_ and some isoprostanes were detected at significant higher levels in the urines of MWS than that of healthy. We added the description in Method section in line 130-133.

6. The urine samples were presumably collected from trays below the cages. One would assume that the trays would contain both urine and feces. It is not clear how the authors conclude that the metabolites detected were only urinary.

**Response:** We collected urinary samples after we visually confirmed that no feces were contaminated. We added the description in Method section in Table 1 and line 104-105.

**Reviewer #2:** Yamazaki et al reported a distinct profile of lipid metabolites in the urine of MWS. Authors identified increased 48 metabolites belonging to AA- and EPA-derived metabolites in the urine of MWS compared to healthy (N=3).

1. Please delete Section “Urine samples”, its is written twice in manuscript.

**Response:** We deleted the word, thank you.

2. No statistical analysis was performed if identified metabolites are significantly altered or not. Number of animals in each group is of a BIG concern as 3 animals cannot be used for appropriate sample size for any in vivo studies. Authors are encouraged to increase the animal number in each group and perform statistical analysis. Without such data, this work has no impact.

**Response:** We increased 4 samples for each group and statistically analyzed 7 samples in each group. The statistical difference was determined by Mann-Whitney U test for two-group comparison. As a result, we found that AA metabolites of PGE_2_, PGF_2α_, TXB_2_ and some isoprostanes were detected at significant higher levels in the urines of MWS than that of healthy. We added the description in Method section in line 130-133.

3. One data point from MWS groups seems to be an outlier. Excluding this point, there is not difference in healthy and MWS groups. This point again pointing to increase the animal number in both groups for meaningful interpretation of data.

**Response:** As mentioned in the response to comment 1, we statistically analyzed with N = 7 data including the outlier sample, we found that some particular types of metabolites were detected at significant higher levels in the urines of MWS than that of healthy.

6. If you would like your identity to be revealed to the authors, please include your name here (optional).

Your name and review will not be published with the manuscript.

Reviewer #1: (No Response)

Reviewer #2: no
