# Supplementary material for: The profile of lipid metabolites in urine of marmoset wasting syndrome
Source: PLoS One. 2020 Jun 23;15(6):e0234634. doi: 10.1371/journal.pone.0234634 (PMC7310677; doi:10.1371/journal.pone.0234634)
Supplement: Attachment — Submitted filename: 20200417 Response to reviewers.docx [file pone.0234634.s004.docx]

**The production profile of lipid metabolites in urine of marmoset wasting syndrome**.

**Review Comments to the Author**

**Reviewer #1**: This work deals with PUFA selected metabolites in Marmosets suffering MWS. Because the cause is still unknown the subject should be deemed quite interesting. By comparison to the attached comments to the previous reviews I find the work almost acceptable for PLoS ONE. Unfortunately, we still do not know what is the real cause of MWS but the authors provided some mediators and mechanism for the development of that illness.

Comment 1: Please add clearly in the conclusion that the cause of MWS remains unknown but the paper revealed some mechanisms and mediators of MWS

<Response>

As the reviewer suggested, we added the descriptions in Abstract and manuscript in line 257 as following; Abstract; “In this study, we found some urinary lipid metabolites suspected to be involved in the development of MWS. Although the cause of MWS remains unclear, our findings may provide some insight into understanding the mechanisms of development of MWS.”

line 257; “We found some lipid metabolites which may be involved in the development of MWS. Since MWS is multifactorial disease and the cause of it remains unclear, we need further investigation to reveal a causal relationship. We believe our findings may provide some insight into understanding the mechanisms of development of MWS.”

Comment 2: Lines 79-83: the authors provide information about different types of cages used in the experiment. Please make it clear for the reader when and why such cages were used. I suppose that an individual cage is ideal for urine sampling. If another cages were used, how the urine was sampled? Add appropriate info sentence.

<Response>

Some of the control marmosets were in pair or family cages, while others were in individual cages for research or management purpose (not related to this study). MWS marmosets were in individual cages when their conditions were deteriorating and required intensive care. When the marmoset was in a pair or family cage, the marmoset was temporarily separated from other individuals using separation wall to collect urine samples. In some occasions, even in a pair or family cage, when a marmoset was urinated in front of us, the urine sample was collected immediately. A clean dry tray was placed under the cage. All urine samples were collected only when visually confirmed not to be contaminated with feces, another individual’s urine, or dropped food. After each sample collection, the trays were sterilized, rinsed thoroughly and dried. The urine samples were stored until use at -80°C. We added the descriptions in Methods “Urine Samples” section in line 116.

**Reviewer #2**: Thank you for your interest in MWS, not enough researches are focused on this to find a solution. As much as I enjoyed your paper, I have a number of reservations that need to be addressed before I can recommend it for publishing. Find the notes added to the manuscript for details.

Comment 1: Diarrhea itself does not equal MWS. You need to add a section on how you identified MWS. From the read of your paper, it sounds like you can't say you were looking at MWS but maybe focus on inflammation or chronic diarrhea. If they do have MWS maybe you can present weight loss data.

<Response>

We identified MWS by the major criteria based on Cabana et al 2018; recurrent diarrhea, absence of pathogenic enteric bacteria and endoparasites, resolution of illness despite anti parasitic treatment and/or antibiotics, and persistent weight loss. The veterinarians in each institution assessed the animals and included in this study. All individuals with MWS had persistent weight loss for over six months. We added the descriptions in “Identification of MWS” in Methods section and the maximum body weight in Table 2.

Comment 2: You could do a better literature search, recent papers on MWS have not been included see text for reference.

<Response> We added the paper “Francis Cabana et al., Zoo Biol, 37 (2), 98-106. 2018.” in line 40, 43, 103, and 118.

Comment 3: Can you give us the nutrient concentrations of their diet? How much AA, DHA and EPA are they actually ingesting? As it stands, all your results support is that there is inflammation in animals with diarrhea. You need the diet information to link with AA. If they eat the same diet and same amount of fatty acids, then causation will be somewhere else. You need the diet information to tie this all together or change the scope of your paper to inflammation and diarrhea.

<Response>

We newly added below table as Table 1, describing the concentration of each PUFAs contained in diet. As shown in table, sum of the concentration of n-6 and n-3 PUFAs were comparable in each diet fed marmoset in three institutions. In addition, we fed the same diet to healthy and MWS marmoset in each institution. Thus, the changes in urinary lipid profiles may reflect some inflammation but not the variation of the concentration of PUFAs derived from diet. We added Table 1 and related descriptions in line 90-92 and 161-162.

|  | PUFAs (g/100 g food) | KUPRI | Jikei | CLEA |
| --- | --- | --- | --- | --- |
| n-6 | Linoleic acid | 4.16 (1.66-2.82) | 3.12 (2.5) | 4.70 (1.88) |
|  | Arachidonic acid | N.D. | 0.04 (0.03) | N.D. |
| n-3 | Linolenic acid | 0.24 (0.10-0.32) | 0.31 (0.25) | 0.54 (0.21) |
|  | DHA | 0.11 (0.02-0.05) | 0.35 (0.28) | 0.04 (0.02) |
|  | EPA | 0.08 (0.01-0.03) |  | 0.02 (0.01) |

**Reviewer #3**: I commend the author's efforts to try and shed light on to disease like WMS which remains a mystery even after so many attempts, however I do think there are some major flaws in the design. I do understand that sample sizes can be challenging but in a prevalent colony as described by the owners, a n of 7 for both groups do significantly limit the conclusions that can be drawn. The title says production profile - I would remove production. I do think there are many authors, actually more authors than study subjects and it would be good to know if they satisfied the criteria for authorship.

<Response>

This study was the first trial of focusing on lipid metabolism in MWS marmosets. We understood the limitation of this study by small sample size so that we do not try to conclude the cause of MWS. But instead, we proposed the possibility of the importance of AA in MWS. We believe that it is important to shed light on MWS from various aspects.

As reviewer suggested we removed the “production” from title. And some author did not satisfy the criteria for authorship, their name is listed in Acknowledgement.

Comment 1: Ln24: It is a syndrome and hence more symptoms are needed to justify the syndrome.

<Response>

We added the maximum weight of each marmoset in Table 2. Indeed, they all had persistent weight loss for over six months. We added the section on Identification of MWS in line 102 Methods Urine Samples.

Comment 2: Ln 34: I do not think given the low number of subjects, a substantial conclusion can be got from this.

<Response>

We understand the limitation of this study by small sample size. We added the descriptions indicating that we did not reveal the mechanisms of MWS in this study as in Abstract and line 257 as following.

Abstract; “In this study, we found some urinary lipid metabolites suspected to be involved in the development of MWS. Although the cause of MWS remains unclear, our findings may provide some insight into understanding the mechanisms of development of MWS.” line 257; “We found some lipid metabolites which may be involved in the development of MWS. Since MWS is multifactorial disease and the cause of it remains unclear, we need further investigation to reveal a causal relationship. We believe our findings may provide some insight into understanding the mechanisms of development of MWS.”

Comment 3: Ln 42: I do think it is redundant to have steroid and glucocorticoids in the same line

<Response>

We deleted the “steroid” in line 42.

Comment 4: Ln 72: Sampling method should be elaborated. LN 104; Were there liners? How did you clean them in between. If feces and urine fall on the same surface, how does one confirm there is no residual effect of the feces. How do you know these inflammatory lipids were not from a biofilm on the floor? The trays need to be described?

<Response>

As we described above as response to the reviewer #1 comment 2, some of the control marmosets were in pair or family cages, while others were in individual cages for research or management purpose (not related to this study). MWS marmosets were in individual cages when their conditions were deteriorating and required intensive care. When the marmoset was in a pair or family cage, the marmoset was temporarily separated from other individuals using separation wall to collect urine samples. In some occasions, even in a pair or family cage, when a marmoset was urinated in front of us, the urine sample was collected immediately. A clean dry tray was placed under the cage. All urine samples were collected only when visually confirmed not to be contaminated with feces, another individual’s urine, or dropped food. After each sample collection, the trays were sterilized, rinsed thoroughly and dried. The urine samples were stored until use at -80°C. We added the descriptions in line 116.

Comment 5: Ln 84 onwards: Diet is a huge component of serum and urine lipid profiles. They should have looked at animals on the same diet to see how they compared.

<Response> We newly added below table as Table 1, describing the concentration of each PUFAs contained in diet. As shown in table, sum of the concentration of n-6 and n-3 PUFAs were comparable in each diet fed marmoset in three institutions. In addition, we fed the same diet to healthy and MWS marmoset in each institution. Thus, the changes in urinary lipid profiles may reflect some inflammation but not the variation of the concentration of PUFAs derived from diet. We added Table 1 and related description in line 90-92 and 161-162.

|  | PUFAs (g/100 g food) | KUPRI | Jikei | CLEA |
| --- | --- | --- | --- | --- |
| n-6 | Linoleic acid | 4.16 (1.66-2.82) | 3.12 (2.5) | 4.70 (1.88) |
|  | Arachidonic acid | N.D. | 0.04 (0.03) | N.D. |
| n-3 | Linolenic acid | 0.24 (0.10-0.32) | 0.31 (0.25) | 0.54 (0.21) |
|  | DHA | 0.11 (0.02-0.05) | 0.35 (0.28) | 0.04 (0.02) |
|  | EPA | 0.08 (0.01-0.03) |  | 0.02 (0.01) |

Comment 6: Ln 104: Urines should be replaced with urine samples.

We revised. Thank you.

Comment 7: Ln 104: What was the duration between voiding and urine collection and storage. Volatile lipids can potentially be lost. Marmosets in certain facilities are trained to urinate, was an attempt for this made.

<Response>

Thank you for your advices. As descried in Response 4, the urine samples were collected as soon as they were found and only when they were not contaminated with feces, urine or dropped food. And then, the urine samples were stored until use at -80°C. We revised Methods Urine samples section in line 116.

Comment 8: Ln 111 onwards: What was the normalization technique used? Was the quantity of metabolites normalized to USG or creatinine. There should also be some way to look into if there was active sediment or not, or at least clinical signs or something to substantiate that these mediators were not produced within the urinary tract as a result of inflammation or infection. WMS is syndrome and hence this is important.

<Response>

We corrected the vales of lipid metabolites by creatinine concentration as described in line 149. Before the collection, we confirmed MWS based on the criteria (Cabana et al 2018) including the absence of pathogenic enteric bacteria and endoparasites.

Comment 9: ln 146: This is an over stretch and this conclusion is not substantiated. I do agree with the other reviewers that the CV and the study design and conclusions have severe limitations, this along with small number of marmosets make me question the validity of these findings. While I do understand how difficult it is to work with marmosets, and samples can be scarce, I do not think the current study can be published as is in good faith. Sorry.

<Response>

We understood the limitation of this study with sample size. This study was the first trial of focusing on lipid metabolism in MWS marmosets. We do not try to conclude that this is the cause of MWS, but instead, we proposed the possibility of the importance of AA in MWS. As described in Response to comment 4, we added the descriptions in Abstract and line 257.

Comment 10: 182- urines should be replaced with urine samples.

<Response>

We revised. Thank you.
