# Supplementary material for: The profile of lipid metabolites in urine of marmoset wasting syndrome
Source: PLoS One. 2020 Jun 23;15(6):e0234634. doi: 10.1371/journal.pone.0234634 (PMC7310677; doi:10.1371/journal.pone.0234634)
Supplement: Attachment — Submitted filename: 20200507 Response to reviewers.docx [file pone.0234634.s005.docx]

**The profile of lipid metabolites in urine of marmoset wasting syndrome**.

**Review Comments to the Author**

**Reviewer #1**: In my opinion the authors improved the text according to the questions raised by the reviewers. It is acceptable for publication.

<Comments>

Thank you very much for your time and suggestions.

**Reviewer #3**: Good job on the revisions. Thank you. I do thing this needs some language editing and proof checking. Please check the manuscript for language errors.

<Comments>

Line 45 MWS. There is also no definitive diagnosis method for MWS. insert 'antemortem'

Line 89 Vitamin D. In CLEA, they were fed on 40-50 g CMS-1M (CLEA japan, - J should be uppercase in Japan

Line 95-96 such as fecal condition, appetite, hair condition and movement, and if they were suspected *any problems, they would be **consulted with veterinarians. - Add *to have, ** evaluated by the veterinarian

Line 105-106 and/or antibiotics, and persistent weight loss [2]. The veterinarians in each institution assessed the animals and *included in this study. - *Prior to inclusion in the study

Line 157 We comprehensively measured - delete comprehensively

Line 257 *multifactorial disease and the cause of it remains unclear, - please add a

In all of the below line numbers, I do think they should say, marmosets with MWS, rather than MWS

Line 109 however, that of MWS were around or less than 300 g at urine samples collection. Frequent and recurrent diarrhea, and persistent weight loss for over six months were observed in MWS

Line 116 as healthy (N = 7) in primary care or MWS (N = 7).

Line 172 acid (DHA) in heathy (N = 7) and MWS (N = 7)

Line 175 The urinary levels of arachidonic acid (AA)-derived catalyzed metabolites in heathy (N = 7) and MWS (N = 7). di. dihydro; te, tetranor; COX, Cyclooxygenase; CYP, CYP450 epoxygenase; LOX, lipoxygenase. *p<0.05, **p<0.01 compared to healthy urines.

Line 180 The urinary levels of arachidonic acid (AA)-derived isoprostanes produced by non-enzymatic oxidation in healthy (N = 7) and MWS (N = 7). **p<0.01 compared to healthy urines.

Line 186 metabolite in healthy (N = 7) and MWS (N = 7). *p<0.05 compared to healthy urines. LOX, lipoxygenase; COX, cyclooxygenase; CYP, cytochrome P450.

Line 218 urinary lipid metabolites in MWS. These results may reflect that MWS marmosets may have

Line 222 in plasma, 13,14-dihydro-15-keto derivatives, were increased in the urines of MWS [22]

Line 231 amounts of PGE2, PGF2α and a metabolite or TXA2 were markedly increased in the urines of MWS.

Line 234 ameliorated dextran sodium sulfate–induced colitis in mice [26]. Collins D et al. showed in an in vitro

Line 240 In this study, we found that F2-IsoPs were increased in the MWS urine samples

Line 250 this study, only 5 types of n-3 PUFAs-derived metabolites were constantly detected in MWS urines

The abstract has many language errors

<Responses>

Thank you for your kind checking our language errors. We carefully revised the errors thoroughly manuscript including abstract.
